# Stress and Coping in Youth With Spina Bifida: A Brief Longitudinal Study in a Summer Camp Setting

**DOI:** 10.3389/fpsyg.2021.682169

**Published:** 2021-08-02

**Authors:** Diana M. Ohanian, Tessa K. Kritikos, Olivia E. Clark, Kezia C. Shirkey, Meredith Starnes, Grayson N. Holmbeck

**Affiliations:** ^1^Department of Psychology, Loyola University Chicago, Chicago, IL, United States; ^2^Department of Psychology, North Park University, Chicago, IL, United States

**Keywords:** coping, stress, spina bifida, chronic condition, camp

## Abstract

**Introduction:**

It is well established that youth with chronic conditions experience elevated levels of stress; the manner in which they respond to or cope with this stress is likely to impact both health and psychosocial outcomes. The current study examined stress and coping in youth and young adults with spina bifida (SB) using the response to stress questionnaire-SB version (RSQ-SB; Connor-Smith et al., 2000).

**Methods:**

Data were collected as part of a camp-based psychosocial intervention for children (ages 7–13), adolescents (ages 14–19), and young adults (ages 20–38) with SB. Participants completed the RSQ-SB as well as questionnaires assessing demographics and condition severity. Data were collected prior to camp (T1) and 1 month (T2) after camp ended. Self-report data were collected from adolescents and young adults; parents of children and adolescents reported on their child’s stress and coping. Ratios of primary control coping, secondary control coping, disengagement coping, involuntary engagement, and involuntary disengagement coping were calculated. Descriptive statistics and *t*-tests were utilized to describe coping and stress responses and to determine potential change over time. *T*-tests were also used to compare youth and parent reported coping styles with those of youth with type 1 diabetes (T1D) and sickle cell disease (SCD). Associations between demographic/disease factors and coping styles were also examined.

**Results:**

Parent and youth report indicated that youth with SB tend to use primary control coping. Youth with SB use more primary control coping and less disengagement coping compared to youth with SCD and youth with T1D. Few significant changes in coping were found between T1 and T2. IQ and socioeconomic status were significantly associated with coping styles.

**Conclusion:**

Youth with SB use more primary control coping compared to other coping methods and as compared to other pediatric populations. Future studies should examine mechanisms by which primary control coping is advantageous for youth with SB. Future interventions should be more focused on promoting adaptive coping behaviors and be tailored to developmental age and access to resources.

## Introduction

Spina bifida (SB) is a relatively common congenital birth defect, affecting approximately 3 in every 10,000 births in the United States (Centers for Disease Control and Prevention, 2011). Due to the failed closure of the neural tube in early pregnancy, most individuals with SB must contend with multiple medical complications, including neurogenic bowel and bladder, ambulation challenges, and social and cognitive deficits. Medical complications often require daily medical care tasks (e.g., catheterization) in order to avoid secondary conditions as well as ongoing management by a number of medical providers. With regard to psychosocial challenges, youth with SB report reduced quality of life, lower self-image, internalizing symptoms, and reduced social contact (Kazak and Clark, 1986; Holmbeck et al., 2003; Copp et al., 2015). While there is a paucity of literature examining the impact of stress in SB, youth with SB often face many disease-related stressors. Stressors experienced by youth with SB are varied and may encompass medical, psychosocial, and family factors.

More generally, the larger pediatric literature has consistently demonstrated that youth with chronic conditions experience significantly elevated levels of stress (Compas et al., 2012). Chronic conditions can require a demanding medical regimen, have taxing symptomatology, and can be accompanied by painful or time-consuming medical procedures. In addition, chronic illnesses can result in reduced school attendance and difficulties with social functioning (Alderfer and Kazak, 2006; Boles, 2017). Furthermore, each condition is characterized by different condition-related stressors (Compas et al., 2012); for example, a child undergoing cancer treatment may struggle with stress related to how treatment has altered their appearance (Zebrack and Isaacson, 2012), whereas a child with diabetes may struggle with stress related to their daily self-management tasks (Rechenberg et al., 2017). A child with SB, on the other hand, may struggle with stress related to ambulation and shunt malfunction.

In response to stress, one may experience automatic stress responses, such as emotional and physiological arousal and stress-conditioned behaviors, or one may respond with a coping behavior. Research considers coping to be a controlled and volitional response; coping is purposeful and directed toward addressing the stressor (Compas et al., 2012). Adaptive coping has been shown to result in better psychosocial functioning, stronger quality of life, and better health outcomes in youth with chronic conditions (Grey et al., 2000; Szigethy et al., 2007). The current literature has produced multiple coping frameworks, which are characterized by various subtypes of coping (e.g., problem-focused coping vs. emotion-focused coping, approach vs. avoidance, etc.; Compas et al., 2012). Coping frameworks that are commonly used with pediatric populations often include active or primary control coping, accommodative or secondary control coping, and avoidant or disengagement coping (Walker et al., 1997; Connor-Smith et al., 2000; Compas et al., 2012).

Stress and coping in pediatric chronic illness can often be challenging to assess, especially taking into account different types of stressors that vary in frequency across chronic illness populations. The response to stress questionnaire (RSQ) was developed by Connor-Smith et al. (2000), and is one of the most frequently used measures of coping responses in pediatric chronic illness populations (Compas et al., 2012). The RSQ assesses specific sources of stress and the use of coping strategies, as well as involuntary stress responses pertaining to a specific stressor (i.e., pediatric cancer, SB), regardless of the success of those coping strategies. The RSQ is valid and reliable, has been supported by several confirmatory factor analyses (Compas et al., 2012), and can be easily completed by youth and/or adults who are involved in the stressful situation (Connor-Smith et al., 2000). Responses to the RSQ comprise five factors: primary control coping, secondary control coping, disengagement coping, involuntary engagement, and involuntary disengagement. Researchers can then compute proportion scores for individuals, indicating coping responses most frequently utilized in relation to the stressor in question.

The first and primary dimension of this measure delineates between involuntary and voluntary responses, with involuntary stress responses considered to occur outside of conscious awareness and reflect individual temperament or conditioned responses to stressors (Connor-Smith et al., 2000). Involuntary and voluntary behaviors are then further subdivided into engagement and disengagement responses wherein engagement responses approach or address the stressor and disengagement responses avoid the stressor. Within voluntary engagement responses, there are two coping types: primary control coping and secondary control coping. Primary control coping strategies attempt to alter the stressor itself or one’s emotional response to that stressor, such as emotional regulation, emotional expression, and problem solving. Secondary control coping strategies are strategies that support adaptation to the stressor, such as acceptance, distraction, cognitive restructuring, and positive thinking (Connor-Smith et al., 2000). Disengagement coping strategies, avoid the stressor, and resultant emotional reactions; examples include, denial, avoidance, and wishful thinking. Finally, there are two involuntary domains: involuntary engagement and involuntary disengagement. An individual experiencing involuntary engagement might have intrusive thoughts or ruminate on the stressor. Further, involuntary disengagement is characterized by emotional numbing, inaction, escape, and cognitive interference (Connor-Smith et al., 2000).

The RSQ coping styles have been associated with multiple outcomes in both typically developing (TD) youth and youth with chronic conditions. Indeed, with regard to psychopathology, a 2017 meta-analysis examining coping in childhood and adolescence in response to various stressors [e.g., interpersonal stress, cancer, type 1 diabetes (T1D), etc.] demonstrated that disengagement coping was associated with increased internalizing and externalizing symptoms, whereas both primary and secondary control coping were associated with fewer internalizing and externalizing symptoms (Compas et al., 2017). These coping styles have also been associated with several physical and psychological outcomes in youth with chronic conditions. Indeed, for youth with T1D, use of primary control coping was positively associated with social competence, quality of life, and low HbA1c. Additionally, this study found that secondary control coping was associated with better social competence and quality of life. In contrast, disengagement was associated with lower social competence and higher HbA1c (Jaser and White, 2011). For youth with chronic abdominal pain, use of secondary control coping has been associated with lower levels of somatic complaints and symptoms of depression and anxiety; the inverse relationship was found with disengagement coping in this population (i.e., more disengagement coping was related to worse somatic complaints and increased symptoms of depression and anxiety; Compas et al., 2012). The positive associations between both primary and secondary control coping and healthy adjustment in chronic illness populations are likely a reflection of the fact that most chronic illnesses include both controllable and uncontrollable stressors, with some having fewer controllable stressors vs. others (Compas et al., 2012). Controllable stressors may be best addressed with primary control coping, whereas secondary control coping would be the most effective strategy to address uncontrollable stressors. With respect to SB, use of secondary control coping strategies, such as acceptance, might be more beneficial when faced with the stress of “not being able to do what others can do,” whereas use of primary control coping, such as problem solving, might be more helpful for “parents bugging me about taking care of myself.” On the other hand, disengagement or avoidance of the aforementioned stressors might result in emotional distress, self-isolation, poor peer relations, reduced independence, and improper self-management and medical adherence.

There is currently very limited literature on how youth with SB cope with stress. Of the few existing studies, research has suggested that coping behaviors develop similarly (i.e., coping socialization) in youth with SB and TD youth (McKernon et al., 2001), and that youth with SB and TD youth employ problem-focused coping, an adaptive coping behavior, with similar frequency when asked to think about a stressful peer encounter (Monsen, 1992). Finally, another study found that adults with SB who received an executive functioning (EF) intervention demonstrated significant gains in adaptive coping strategies (decreased avoidant-focused and increased task-focused coping; Stubberud et al., 2014). To our knowledge, no study has examined (1) how youth with SB respond to illness-related stress, (2) if there are differences between how youth with SB and youth with other pediatric conditions respond to illness-related stress, and (3) the effectiveness of a psychosocial intervention that teaches adaptive coping strategies to youth with SB.

Therefore, the first aim of the current study was to examine coping methods and stress responses in youth with SB using the response to stress questionnaire-SB (RSQ-SB), and to compare our study sample’s responses to that of other pediatric populations that contend with similar illness-related stressors (e.g., samples with self-management concerns, complex medical regimens, or neurocognitive difficulties). For this study, we chose to examine differences between youth with SB and youth with T1D and sickle cell disease (SCD). Similar to SB, both of these populations contend with complex medical regimens. Specifically, the treatment regimen for T1D typically requires checks for blood glucose levels, dietary restrictions, insulin injections, and other related medical tasks (American Diabetes Association, 2016). Likewise, youth with SCD often manage medication regimens or adhere to complex therapies (e.g., blood transfusions for anemia) as well as engage in preventative behaviors to reduce the likelihood of having a pain crisis (i.e., staying hydrated, avoiding sudden temperature changes; Center for Disease Control and Prevention, 2020). Further, both youth with SB and SCD struggle with neurocognitive deficits. Youth with SCD, regardless of cerebral infarct history, typically have lower IQs compared to their TD peers (Schatz et al., 2002; Prussien et al., 2018). Likewise, while intellectual functioning in youth with SB tends to fall in the low average to average range, there is significant variability within the condition; indeed, 20–25% of individuals with SB-myelomeningocele and hydrocephalus are estimated to have an intellectual disability (Copp et al., 2015). Regarding cognitive functioning in T1D, although there is some evidence for cognitive differences between youth with T1D and TD youth (Kirchhoff et al., 2017), particularly in cases of poorly controlled insulin, these are not often considered to be clinically significant. Further, cognitive differences may not be as prominent in youth with SCD vs. SB. Therefore, the unique cognitive profile in SB should be considered when examining comparisons with T1D and SCD youth. Nevertheless, given similarities in the condition-related stressors amongst these populations, comparisons between SB, and T1D and SCD will provide valuable insights into the nature of coping in SB.

The second aim of this study was to examine relations between stress and coping responses and condition and demographic factors in this population. While there are mixed findings with regard to demographic factors and coping, some studies have found that younger children employ more primary control vs. secondary control coping strategies, a difference which may be related to certain cognitive skills needed to engage in secondary control coping (Weisz et al., 1994; Thomsen et al., 2002). Interestingly, other studies have not found any age-related differences (Compas et al., 2012). As previously noted, intellectual functioning is highly variable among youth with SB. Thus, many children with SB have different developmental ages vs. their chronological age as a result of this condition (Copp et al., 2015). Furthermore, it is also important to consider the role of EF in relation to coping, as EF is challenging for youth with SB (Brown et al., 2008) and some studies have found associations between EF skills and greater use of both primary and secondary control coping (Compas et al., 2012). Therefore, it was hypothesized that younger participants will use more primary control strategies compared to older participants. Further, youth with lower levels of intellectual functioning were expected to use both primary and secondary control coping less compared to youth with higher intellectual abilities.

It is also important to examine youth coping responses longitudinally and determine what types of interventions promote positive coping in this population. Therefore, the third aim of the current study was to examine potential differences in coping behaviors after attending a camp-based psychosocial intervention. Camp Independence is a summer camp for children, adolescents, and young adults with SB. It is an accessible camp that promotes recreation as well as social and independence skill building. At this camp, youth enjoy typical summer camp experiences (e.g., swimming, archery) in a safe environment with same age peers with SB. In addition to engaging in recreation activities, youth also participate in a psychosocial intervention for 1 h per day, which addresses independence, social skills, emotional wellness, and self-care. This intervention is tailored to three different age groups (i.e., 7–13; 14–19; and 20+). The main themes of these 4 days include (1) taking care of your relationships, (2) self-care, (3) living with SB, and (4) taking care of SB. Previous studies have found Camp Independence to result in improved management of SB responsibilities and increased independence in SB task management 1 month post-intervention (O’Mahar et al., 2010; Holbein et al., 2013). More generally, medical camps have been found to lead to improvements in psychosocial functioning, including improvements in self-esteem, emotional functioning, and coping (Hunter et al., 2006; White et al., 2016). Given the aforementioned findings regarding the success of Camp Independence and other medical camp interventions in improving youth psychosocial functioning, as well as Camp Independence’s focus on increasing multiple forms of adaptive coping (i.e., both primary and secondary), it was hypothesized that youth would report an increase in primary and secondary control coping strategies post-intervention.

## Materials and Methods

### Participants

Participants consisted of campers aged 7–38 years, who attended a weeklong overnight summer camp in 2019. Camp Independence exclusively serves individuals with SB, is located in northern Illinois, and is funded in part by the Illinois Spina Bifida Association (ISBA). Camp Independence is also associated with the YMCA. Camp/study inclusion and exclusion criteria were as follows: individuals needed to be at least 7 years old to apply and those with severe allergies or unpredictable health conditions (e.g., uncontrollable seizures) were ineligible. Camp sessions were separated into three age groups: Group A (children, age 7–13; *M* = 10.94, SD = 1.73), Group B (adolescents, ages 14–17; *M* = 15.35, SD = 1.35), and Group C (young adults, ages 18–38; *M* = 26.96, SD = 5.60). The current study used data from two time points: Time 1 (T1; pre-intervention) and Time 2 (T2; 1-month follow up post-intervention). For this study, data were collected from parents of campers in groups A and B and from campers in groups B and C at both time points. All campers were approached to participate in the study; however, it was not required that everyone at camp participate in this study. Further, regardless of whether or not the camper decided to participate in the study and fill out questionnaires, all campers attended the daily-intervention workshop, as it was embedded into the camp programming.

### Procedure

This study was approved by the Institutional Review Board at Loyola University Chicago. Participants were recruited *via* flyers given at regularly scheduled doctor’s visits as well as print and online information disseminated by the ISBA. Questionnaires, and assent and consent forms were mailed to enrolled campers’ homes prior to the start of camp. Parent consent and camper consent/assent were completed either before the beginning of camp *via* mailed forms or were completed on the first day of camp. With regard to the timing of the questionnaires, questionnaires at T1 were completed prior or on the first day of camp, and T2 questionnaires were completed 1 month after the end of camp.

At T1, parents and campers completed questionnaires assessing demographic and medical information as well as various aspects of camper functioning. Throughout camp, trained research assistants administered brief cognitive assessments to obtain an estimate of intellectual functioning. At T2, parents and campers again completed questionnaires that examined the same domains of functioning as were assessed at T1 and requested feedback regarding the intervention and camp activities in general.

### Intervention

A description of the 2010 version of the intervention can be found in Holbein et al. (2013). Since that time, the programming has undergone minor changes and improvements. The intervention includes 1-h daily workshops, which occurred during 4 days of the weeklong camp program. Each day of the intervention had its own theme related to promoting better overall psychosocial functioning. The intervention was composed of the following themes (1) taking care of your relationships, (2) taking care of yourself, (3) living with SB, and (4) taking care of SB. Further, this workshop was tailored to be developmentally appropriate with regard to the three different age groups. For example, the intervention involved more games and examples related to siblings for the younger age groups, whereas, with the older group, the intervention was more didactic in nature and included programming related to, for example, romantic relationships. Day 1 focused on social skill building, Day 2 focused on self-care, relaxation, and coping, Day 3 focused on discussing SB with others, and Day 4 focused on developing SB knowledge, and taking responsibility for SB tasks. Day 2, the focus of this study, involved affect recognition, developing positive coping skills and avoiding negative stress responses, as well as psychoeducation regarding recognizing depression and anxiety and how to ask for help.

Although this invention was not created specifically to promote coping skills, it taught both primary control coping and secondary control coping. Such coping skills were predominately derived from cognitive behavioral, dialectical behavioral, and mindfulness related orientations. Primary control coping skills included problem solving and emotion regulation. Problem solving was typically oriented around self-management (e.g., catheterization) and communication skills (talking with friends). Emotion regulation skills included teaching distress tolerance skills (Dialectical behavioral therapy), specifically the Soothe with Six Senses lesson. Secondary Control coping skills included distraction, cognitive restructuring, and positive thinking. Youth were taught to identify pleasurable activities (e.g., spending time with friends, exercising, relaxation) and engage in those activities when feeling sad or anxious (i.e., distraction). Relaxation strategies included deep breathing, guided visualization, and progressive muscle relaxation. With regard to cognitive restructuring, youth were taught how to make coping statements/cards (i.e., “This is hard but I have gotten through it before!”) With regard to positive thinking, youth were also taught that when they felt down to “look for the positives,” such as remembering three positive things that happened that day prior to experiencing the stressor. Finally, we also provided psychoeducation on the negative impact of disengagement coping and provided the aforementioned alternatives to encourage the use of adaptive coping strategies.

### Measures

#### Medical and Demographic Variables

Parents and young adults completed questionnaires assessing demographic information and medical history. Demographic information included child/adolescent/young adult age, gender, race, ethnicity, and family household income. Medical information included lesion level, type of SB, shunt status, and ambulation method. Participants indicated their family household income using a 21-point scale; the scale ranged from under $10,000 per year to over $200,000 per year, using increments of $10,000.

#### Response to Stress Questionnaire

Parents, adolescents, and young adults completed the RSQ-SB version at T1 and T2 (Connor-Smith et al., 2000). This questionnaire measures coping and involuntary stress responses. It has been adapted to capture the experience of coping and stress for a variety of populations (e.g., pediatric cancer, autism, school stress). It has been translated into four languages, including Spanish; the Spanish and English versions of this questionnaire were used for this study. Similar to other forms of the RSQ, the RSQ-SB starts with a checklist of stressors related to a specific domain of stress which was, in this case, SB. With those stressors in mind, the participant is then asked to indicate how often they use certain coping methods or how often they experienced an involuntary stress response, using a Likert scale (range 1–4). Parents reported on their *child’s* use of coping methods or how often their *child* experienced an involuntary stress response. In addition to the Likert ratings, participants were also often asked how they used a given strategy (e.g., after rating how often they “let someone or something know how I feel” they are asked to “check all that you talk to,” which includes parent, teacher, friend, God, pet, etc.). The coping and stress responses fall into five factors: primary control coping, secondary control coping, disengagement, involuntary engagement, and involuntary disengagement. Factor ratios, or how much a person engages in behaviors that fall into a specific factor, were calculated by dividing the score of each factor by the total score for the RSQ. This methodology is intended to control for response bias and individual differences in the rate of item endorsement (Vitaliano et al., 1987).

#### Cognitive Ability

To estimate cognitive ability, the Vocabulary and Matrix Reasoning subtests from the Wechsler Abbreviated Scale of Intelligence (WASI; Wechsler, 1999) were administered to campers. These two subtests yield an estimated Full-Scale IQ (FSIQ). For campers who had completed this testing within the past 2 years while participating in the camp intervention, their prior WASI score was extracted for analyses.

#### Data Analyses

Descriptive statistics, including mean and standard deviation (SD), were used to address Aim 1 of this study. These analyses included the means and SDs of parent and youth report of the ratios of coping styles. Further, to address Aim 1 we also chose two study samples (T1D and SCD) against which to compare use of primary control coping, secondary control coping, and disengagement coping. Means, SDs and sample size were extracted from Jaser et al. (2017) and Prussien et al. (2018) to conduct unpaired samples *t*-tests. For comparisons, we used the current study’s T1 data. In Jaser et al. (2017), 117 youth (ages 10–16) with T1D completed the self-report RSQ, and in Prussien et al. (2018), 44 parents of youth with SCD (ages 6–16) completed the RSQ-parent report about their child. As previously noted, this choice was guided by an examination of current literature on coping in pediatric populations to assess which populations struggle with similar concerns to that of youth with SB. We also examined the stressors listed in the RSQ for these populations and determined that there were many similarities in item content [e.g., “dealing with diabetes care,” “having to go to the hospital or clinic so often (for Sickle Cell Disease care)”].

However, it is also important to note several differences in the demographics across these samples. Specifically, there is a larger age range in the current study’s sample vs. both the T1D sample and the SCD sample. Sample race and ethnicity could not be directly compared given limited information in both of the comparison samples. However, race was predominately White, non-Hispanic, in Jaser et al. (2017; T1D) and Black in Prussien et al. (2018; SCD; participants in the study identified as African American); race was predominately White, non-Hispanic, in the current study sample. With regard to family income, in Jaser et al. (2017; T1D) family income was controlled for in analyses but was not explicitly reported. Instead they reported that their study sample had a “fairly high socioeconomic status and income.” On the other hand, the majority of the SCD sample (67.4%) in Prussien et al. (2018) reported family income to be less than $50,000 per year. Therefore, as our sample’s mean income was around $95,100 per year (SD = $49,100), comparisons should take into account differences in socioeconomic status. Further, regarding intellectual functioning, there were no relevant data on the T1D sample. However, as previously noted, cognitive functioning is not typically reduced in T1D; therefore, we would expect intellectual functioning in that sample to be higher. Further, while it was not possible to directly compare intellectual functioning between the SB sample and the SCD sample, no statistical differences were detected [*t*(114) = 1.96, *p* > 0.05] between our sample’s FSIQ score (*M* = 87.26, SD = 18.42, range: 55–136) and the verbal comprehension standard score (*M* = 93.39, SD = 12.08, range = 62–123) reported in Prussien et al. (2018). Finally, it should also be noted that youth with SB may struggle with difficulties seen in those with intellectual and physical disabilities, as well as chronic health conditions. However, SB is often considered a “snowflake” condition (i.e., symptomatology in SB is quite variable; Stiles-Shields et al., 2019), therefore, these comparison samples were determined to be *appropriate* comparison groups rather than perfect comparison groups.

To address Aim 2, bivariate Pearson correlation coefficients were computed to examine associations between the demographic/disease factors and coping. Due to differences in sample size, independent-samples Kruskal–Wallis tests were also conducted to examine associations between coping, and SB type, shunt status, lesion level, and, race and ethnicity. To address Aim 3 paired sample *t*-tests were performed to compare parent report of coping and stress response style at T1 vs. T2 and youth report of coping and stress response style at T1 vs. T2 (T2 = post-camp intervention).

## Results

### Preliminary Analyses

A total of 77 families provided RSQ data at T1, including 46 parents and 45 youth. There were 15 families for whom we had both parent and youth RSQ data, and therefore we used a composite score based on both parent and youth report. At T2, a total of 48 families provided RSQ data, including 29 parents and 25 youth. There were six families for whom we had both parent and youth RSQ data; therefore, we again used a composite score based on both parent and youth report. Other studies using the RSQ have similarly adopted this technique of collapsing across reporters to reduce the number of analyses (see Vreeland et al., 2019). With regard to attrition, there were no statistically significant differences between those families who participated at T2 (*n* = 48) and those who did not participate at T2 (*n* = 29) with regard to child age, child sex, child IQ, child lesion level, family income, or primary or secondary control coping ratios.

Among participating families at T1, most youth were female (*n* = 44, 57.9%) and White (*n* = 57, 75.0%). The majority of parents were female (*n* = 38, 82.6%). Youth ranged in age from 7 to 38 years old, with a mean child age of 18.43 (SD = 8.6) and had an average IQ of 87.26 (SD = 18.42, range: 55–136). Yearly family income averaged approximately $95,100 per year (SD = $49,100). In terms of medical characteristics, youth most often had myelomeningocele SB (*n* = 63, 82.9%), lumbar lesion levels (*n* = 41, 53.9%), and a shunt present (*n* = 58, 76.3%). See Table 1 for additional descriptive information regarding demographic and medical characteristics.

**TABLE 1:** Descriptive demographic and medical information for sample, *n* = 76.

Variable	*n*	%
**Child sex**		
Female	44	57.9
Male	30	39.5
Missing	2	2.6
**Parent sex^a^**		
Female	38	82.6
Male	4	8.7
Missing	4	8.7
**Child race/ethnicity**		
White	57	75.0
African American	5	6.6
Hispanic/Latino	9	11.8
Asian American	1	1.3
Biracial (African American and White)	1	1.3
Missing	3	3.9
**Child type of SB**		
Myelomeningocele	63	82.9
Occulta	2	2.6
Lypomeningocele	1	1.3
Meningocele	2	2.6
Not sure	8	10.6
**Child lesion level**		
Sacral	13	17.1
Lumbar	41	53.9
Thoracic	3	3.9
Not sure	18	23.7
Missing	1	1.3
**Shunt status**		
Present	58	76.3
Not present	16	21.1
Missing	2	2.6

	***M* (SD)**	***n***

Child age (range: 7–38 years)	18.43 (8.6)	75^b^
Child IQ (range: 55–136)	87.26 (18.42)	72^b^
Yearly family income (range: 2–21)	10.51 (5.91)	41^a^

*Yearly family income was reported on a 21-point scale, from <$10,000 per year to >$200,000 per year, with each point on the scale representing increments of $10,000. For this sample, family income ranged from $10,000 to >$200,000+ per year with a mean of ∼$95,100 and a standard deviation of ∼$49,100.*

*^*a*^Sample size is reduced for these characteristics because we only obtained parent report for these characteristics, and approximately half of the sample was over the <18 without a parent-report version.*

*^*b*^Sample size is reduced for these characteristics because of missing data/assessments.*

### Aim 1: Descriptive Information Regarding Coping Measure and Comparison to Other Pediatric Samples

Descriptive information (including means and SDs) for each subscale of the RSQ at T1 and T2 by reporter (i.e., parent and youth) are provided in Table 2. At T1, the ratio of primary control coping was greatest and significantly higher than the ratio of secondary control coping for both parents [*t*(45) = 2.68, *p* = 0.010] and youth [*t*(44) = 2.05, *p* = 0.047]. While the tendency to engage in primary control coping vs. secondary control coping was maintained for youth at T2 [*t*(24) = 2.19, *p* = 0.04], there was no statistically significant difference in parent endorsement of primary vs. secondary control coping at T2 [*t*(28) = 1.07, *p* = 0.29]. The ratio of primary control coping was also higher than the ratio of disengagement coping for both parents [*t*(45) = 5.975, *p* < 0.001], and youth [*t*(44) = 5.262, *p* < 0.001]. This tendency to engage in primary control coping vs. disengagement coping was maintained at T2, as reported by both youth [*t*(24) = 4.058, *p* < 0.001] and parents [*t*(28) = 3.065, *p* = 0.005].

**TABLE 2:** Parent- and youth-reported RSQ scores at T1 and T2.

Variable	Time 1		Time 2			
	*M*	SD	*M*	SD	*t* (df)	*p*-Value
**Parent report**						
Ratio primary control coping	0.41	0.35	0.38	0.31	0.62 (28)	0.54
Ratio secondary control coping	0.21	0.23	0.26	0.20	−1.65(28)	0.11
Ratio disengagement coping	0.07	0.07	0.13	0.17	−2.14(28)	0.04
Ratio involuntary engagement coping	0.23	0.24	0.14	0.10	1.63 (28)	0.11
Ratio involuntary disengagement coping	0.09	0.09	0.09	0.07	−0.09(28)	0.93
**Youth report**						
Ratio primary control coping	0.35	0.26	0.41	0.34	−1.21(24)	0.24
Ratio secondary control coping	0.22	0.19	0.20	0.14	1.44 (24)	0.16
Ratio disengagement coping	0.10	0.10	0.09	0.07	−0.37(24)	0.71
Ratio involuntary engagement coping	0.20	0.17	0.16	0.12	1.17 (24)	0.26
Ratio involuntary disengagement coping	0.14	0.14	0.14	0.12	−0.24(24)	0.82

*M, mean; SD, standard deviation.*

Descriptive information and results from unpaired samples *t*-tests comparing youth with SB to youth with SCD and T1D can be found in Table 3 (Note: all SB data were from T1). With regard to comparisons with SCD, parents of youth with SB reported a significantly higher ratio of primary control coping [*t*(88) = 4.15, *p* = 0.00] and significantly lower ratio disengagement coping than parents of youth with SCD [*t*(88) = 6.39, *p* = 0.00; Prussien et al., 2018]. There was no statistically significant difference when comparing the ratio of secondary control coping in parent report of youth with SB vs. parent report of youth with SCD [*t*(88) = 1.67, *p* = 0.10]. With regard to comparisons with T1D, youth with SB reported a significantly higher ratio of primary control coping vs. youth with T1D [*t*(160) = 6.49, *p* = 0.00], and significantly lower ratios of secondary control coping [*t*(160) = 2.63, *p* = 0.01] and disengagement coping [*t*(160) = 3.91, *p* = 0.00] vs. youth with T1D (Jaser et al., 2017).

**TABLE 3:** Parent- and youth-reported RSQ scores for youth with SB, SCD, and TD1.

Variable	SB		SCD			
	*M*	SD	*M*	SD	*t* (df)	*p*-Value
**Parent report**						
Ratio primary control coping	0.41	0.35	0.19	0.03	4.15 (88)	0.00
Ratio secondary control coping	0.21	0.23	0.27	0.06	1.67 (88)	0.10
Ratio disengagement coping	0.07	0.07	0.14	0.02	6.39 (88)	0.00

	**SB**		**TD1**			
	***M***	**SD**	***M***	**SD**	***t* (df)**	***p*-Value**

**Youth report**						
Ratio primary control coping	0.35	0.26	0.19	0.04	6.49 (160)	0.00
Ratio secondary control coping	0.22	0.19	0.27	0.05	2.63 (160)	0.01
Ratio disengagement coping	0.10	0.10	0.14	0.03	3.91 (160)	0.00

*SCD, sickle cell disease; TD1, type 1 diabetes; *M*, mean; SD, standard deviation.*

### Aim 2: Associations Between Demographic and Medical Characteristics and Coping

Table 4 displays correlation results between child demographic and medical characteristics and composite coping scores at T1 and T2. Child age was significantly correlated with involuntary disengagement in T2, such that older children were more likely to engage in involuntary disengagement than younger children (*r* = 0.32, *p* = 0.02). Child IQ was significantly associated with the ratio of primary control coping at T2, such that lower IQ was associated with more primary control coping (*r* = −0.31, *p* = 0.03). IQ was also associated with the ratio of secondary control coping at T2, such that higher IQ was associated with more secondary control coping (*r* = 0.40, *p* = 0.005). Finally, family income was significantly associated with the ratio of secondary control coping at T1, such that higher family income correlated with more secondary control coping (*r* = 0.48, *p* = 0.001). There were no significant associations between child gender, SB type, shunt status, or lesion level and any of the coping subscales.

**TABLE 4:** Correlations between composite RSQ scores and demographic and medical variables.

Variables	1	2	3	4	5	6	7	8	9	10	11	12	13	14
1. Child age	1													
2. Child gender	–0.09	1												
3. Child IQ	−0.25*	–0.06	1											
4. Family income	0.22	0.14	0.04	1										
5. T1 primary control	–0.08	0.21	0.03	–0.26	1									
6. T1 secondary control	0.15	–0.11	–0.09	0.48**	−0.57**	1								
7. T1 disengagement	0.22	–0.09	–0.06	0.12	−0.43**	0.15	1							
8. T1 involuntary engagement	–0.21	–0.11	0.11	–0.13	−0.51**	–0.17	–0.08	1						
9. T1 involuntary disengagement	0.16	–0.10	–0.06	0.13	−0.45**	0.00	0.27*	–0.03	1					
10. T2 primary control	–0.04	–0.09	−0.31*	–0.01	–0.11	–0.16	–0.16	0.31*	0.05	1				
11. T2 secondary control	0.02	0.04	0.40**	–0.05	0.03	0.23	0.09	–0.24	–0.04	−0.72**	1			
12. T2 disengagement	–0.15	0.07	0.08	–0.22	0.16	–0.05	0.12	–0.19	–0.09	−0.49**	–0.00	1		
13. T2 involuntary engagement	0.01	0.05	0.26	0.30	0.08	0.12	–0.01	–0.23	0.01	−0.79**	0.56**	0.08	1	
14. T2 involuntary disengagement	0.32*	0.09	0.00	0.21	–0.03	0.09	0.24	–0.13	0.03	−0.61**	0.23	0.06	0.55**	1

*Correlation is significant at the 0.05 level.

**Correlation is significant at the 0.01 level.

### Aim 3: Examine the Utility of a Camp Based Psychosocial Intervention With Regard to the Promotion of Healthy Coping Behaviors

According to parent-report, ressults showed no significant differences in the ratio of primary control coping, secondary control coping, involuntary engagement coping, and involuntary disengagement coping from T1 to T2. However, there was a significant increase in the ratio of disengagement coping from T1 to T2 [*t*(28) = −2.14, *p* = 0.04]. According to youth report, results showed no significant differences across all five coping and stress response styles between T1 and T2 (Table 2).

## Discussion

Research has repeatedly documented the advantages of adaptive coping in pediatric populations. To our knowledge, no study to date has examined how youth with SB cope with the stressors that accompany this condition. Therefore, the purpose of this study was to gain foundational knowledge regarding how youth with SB cope with disease-related stressors. We also aimed to determine whether youth with SB cope in a manner similar to youth with other chronic conditions (i.e., T1D and SCD). Further, given the complexity of this condition we sought to understand relations between coping behaviors and condition-related factors (e.g., lesion level, shunt status, etc.). We also examined relations between demographic factors (e.g., age, gender, SES, etc.) and coping in this population. We hypothesized that younger children would engage in more primary control coping compared to older children. Further, we hypothesized that those with lower intellectual functioning would report using less primary and secondary control coping. Finally, we examined the utility of a brief psychosocial intervention within a camp environment for promoting positive coping behaviors 1-month post-intervention. It was hypothesized that use of both primary and secondary control coping would increase post-intervention. We addressed these aims using the recently created SB version of the RSQ, a well-validated and commonly used measure of coping, particularly for pediatric populations (Compas et al., 2012).

Regarding our first objective, examining how youth with SB respond to illness-related stress, results revealed that youth with SB tend to utilize primary control coping strategies when addressing SB-related stress. Indeed, both parent and youth report indicated that youth with SB used primary control coping strategies at T1 significantly more than secondary control coping and disengagement coping. These results suggest that when confronted with a stressor youth with SB often try to alter the stressor itself or their emotional response to that stressor (i.e., emotional regulation, emotion expression, problem solving). This is an interesting finding given the intractable nature of many stressful aspects of SB (e.g., self-management, having to go to clinic often). However, while problem solving may not be always *as* useful for youth with chronic conditions, emotion regulation or expression might be a useful strategy for youth with SB. Further, these findings overall demonstrate that youth with SB tend to use an *adaptive* coping strategy when faced with illness-related stress, which represents an area of strength. Nevertheless, we must also consider the fact that, with regard to parent report, primary control coping may be more easily recognized in their children vs. secondary control coping. Indeed, secondary control coping involves more internal processes, which may not be readily seen by parents observing their child’s response to stress.

Results also suggested that youth with SB may cope differently with illness-related stressors compared to youth with other chronic conditions. Indeed, according to parent report, youth with SB utilize more primary control coping and less disengagement coping than youth with SCD (Prussien et al., 2018). Further youth self-report indicated that youth with SB may utilize more primary control coping, and less secondary control coping and disengagement coping than youth with T1D (Jaser et al., 2017). Overall, these results further demonstrate that youth with SB have a strong tendency to use primary control coping more than other methods, even in comparison to other pediatric populations that contend with similar illness-related stressors.

Our second objective was to examine associations between coping and a variety of medical and demographic characteristics. Interestingly, this study did not find any significant associations between coping and disease factors. This finding may be due, in part, to the homogeneity of our sample with regard to certain disease characteristics (e.g., most of the sample had myelomeningocele SB and a shunt). With regard to demographic factors, contrary to hypotheses, we found no differences between younger and older youth with regard to the use of primary and secondary control coping. However, results did indicate that older participants were more likely to engage in involuntary disengagement (e.g., emotional numbing, escape) than younger participants. This may suggest that as youth become older, become more aware of disease stressors, and become more responsible for their care, they become emotionally overwhelmed, and unintentionally engage in maladaptive stress responses. It will be important for future studies to explore this finding further, as disengagement coping has been found to be associated with increased internalizing symptoms (Compas et al., 2017). Therefore, this finding may highlight an important area for intervention, wherein the teaching of positive coping skills could mitigate risks for developing depressive and anxious symptomatology, which are associated both with this developmental period (Hyde et al., 2008) and having SB (Holmbeck and Devine, 2010).

Our hypothesis, that those with lower intellectual functioning would use less primary and secondary control coping, was partially supported. Specifically, lower IQ was significantly associated with more use of primary control coping strategies (in contrast to our hypothesis), whereas higher IQ was associated with using more secondary control coping strategies (in line with our hypothesis). Compas et al. (2012) suggested that older age may be related to more use of secondary control coping due to the cognitive demands of reappraisal, acceptance, etc. Therefore, this finding may reflect coping differences in relation to developmental age. It may also be related to the EF deficits commonly found in SB (Brown et al., 2008). Strong EF has been found to be associated with more use of secondary control coping in multiple pediatric populations (Campbell et al., 2009; Desjardins et al., 2018). Further, another study found that stronger verbal comprehension was related to increased use of secondary control coping in youth with SCD (Prussien et al., 2018). They hypothesized that cognitive reappraisal relies on internal self-speech, which is more easily accessible to those with stronger verbal abilities. While youth with SB have relative strengths in verbal abilities compared to non-verbal abilities (Dennis and Barnes, 2010), they tend to struggle with more complex verbal skills that involve integrating information and applying previously acquired knowledge (Dennis et al., 2006), skills that likely underlie the ability to engage in secondary control coping.

Moreover, higher family income was positively associated with secondary control coping. Research on associations between SES and different coping styles have produced mixed results. One study found that cognitive coping strategies, a core component of secondary control coping, were associated with more symptoms of anxiety and depression in a lower SES group, potentially indicating that these strategies may not be as effective in high stress environments (Perzow et al., 2021). However, other studies have found no differences in coping strategies in relation to SES (Gage-Bouchard et al., 2013). Still, others have argued that secondary control coping strategies may be particularly helpful for low SES adolescents, due to the unchangeable nature of the stressors they face (DeCarlo Santiago and Wadsworth, 2008). Overall, future research is necessary to understand the relationship between SES and coping in this population in order to inform the development of effective interventions.

The final aim of the study was to determine the utility of a weeklong camp-based intervention in promoting adaptive coping. In contrast to our hypothesis, no differences were found in terms of primary or secondary control coping for both parent and youth report from T1 to T2. According to parent report, the ratio of disengagement coping appeared to increase significantly from T1 to T2. Still, this was a relatively small change and indicates that coping was generally stable over time. Therefore, this intervention, which has demonstrated positive effects on independence skills, and individual self-care and social goals (Holbein et al., 2013), did not significantly improve coping style. This finding likely reflects the fact that the intervention at Camp Independence is not a “coping intervention”; indeed, adaptive coping is only addressed on 1 day of this weeklong intervention. Further, this finding may again point to the difficulties in using parent report for assessing coping in a child; it may be quite difficult for a parent to recognize change in their child’s chosen coping strategies. These findings also support past research that has found coping styles to be generally stable over time (Kirchner et al., 2010; Shirkey et al., 2011).

### Limitations

This study had several strengths including the use of a well-validated measure of coping that had yet to be used in this population, a large age range, multiple reporters, and a longitudinal design. Nevertheless, there were several limitations that should be noted. First, the study was overall descriptive in nature. Second, the study’s sample size was small and predominately White, which limited our ability to test the generalizability of our findings. Further, our ability to accurately assess potential differences in coping styles with regard to certain demographic variables (i.e., age, IQ, income) was also hindered by a further reduced sample size due to missing data. Attrition from T1 to T2 is another important limitation to note. Our reduced sample size likely impacted on our longitudinal analyses, and left this study vulnerable to Type II error. Further, the drop in the sample size due to attrition was primarily amongst the youth self-report vs. parent report. Indeed, as previously noted, there are some concerns with parents ability to report on their child’s coping; therefore, the loss of self-reported coping from T1 to T2 likely impacted our ability to detect statistically significant differences in coping over time. Further, our data were collected 1 month apart, which may not have been enough time to detect meaningful changes in coping. Future studies should repeat these analyses using a longer-term longitudinal design. Finally, the intervention discussed in this study only had 1 day dedicated to teaching about adaptive coping strategies; therefore, the non-significant findings with regard to change over time in coping style should be interpreted with caution. Such a lack of change in coping likely reflects the limitations associated with this intervention in terms of improving coping skills, rather than a reduced potential for skill improvement with a more focused intervention in this population.

## Conclusion

The results of this study have several important clinical implications for promoting the psychosocial wellbeing of youth and young adults with SB. This was the first study to systematically examine coping styles in youth with SB. Research has consistently demonstrated that the use of adaptive coping strategies leads to improvement in social, emotion, and medical outcomes in pediatric chronic illness populations (Compas et al., 2012). This study was able to highlight areas of risk and resilience that can be built upon to create effective and targeted interventions. Results from this study indicated that youth with SB predominately use primary control coping strategies. Further, our findings suggest that youth with SB respond to illness-related stressors in a different manner compared to other pediatric populations (SCD and T1D). Specifically, youth with SB appear to use more primary control coping and less disengagement coping compared to SCD and T1D, and less secondary control coping compared to youth with T1D. Therefore, this study identified that youth with SB have a tendency to utilize an adaptive coping strategy, highlighting a significant strength that can be built upon in the context of clinical intervention (e.g., focusing on primary control coping in therapy). Future studies should examine the effectiveness of clinical interventions that promote the use of primary control coping (e.g., modules of CBT such as problem solving) for reducing illness-related stress in youth with SB. Supporting the use of primary control coping may contribute to improving psychosocial and medical outcomes in this population.

Nevertheless, given that there are many intractable stressors in this condition, future studies should also examine the utility of using primary control coping in this population (i.e., some SB-related stressors may be less responsive to primary control coping) in order to develop interventions that utilize this strength (i.e., tendency toward primary control coping) when appropriate and teach secondary control coping strategies for stressors that are less likely to respond to primary control coping. Further, coping strategies were associated with intellectual functioning, such that lower IQ was associated with more frequent use of primary control coping and higher IQ was associated with more frequent use of secondary control coping. Therefore, it will be important to consider developmental age in addition to the nature of the stressor itself when creating clinical interventions. For example, for youth with lower IQ, it may be more effective to focus on promoting primary control coping strategies, such as emotion regulation, when faced with unchangeable stressors vs. acceptance, which is a more complex construct/strategy. Further, when one teaches secondary control coping to youth with lower cognitive functioning, it may be beneficial to focus on strategies that are less complex (distraction vs. cognitive restructuring). Relatedly, as EF deficits are common in this population, increased structure when teaching adaptive coping is essential. Helpful strategies may include: reminders on the patient’s phone, visual cue cards to prompt the patient to engage in coping strategies, having a simple menu of coping skills ready and accessible to use, involvement of parents in therapy to facilitate the practicing of coping skills in between sessions. This study also found that higher SES was associated with more secondary control coping. Future studies should continue to examine the relationship between SES and coping styles in this population in order to develop actionable interventions for youth from diverse socioeconomic backgrounds. Finally, this study found that coping styles in youth with SB did not change significantly over time, further demonstrating the consistency of coping styles in pediatric populations.

## Data Availability Statement

The raw data supporting the conclusions of this article will be made available by the authors, without undue reservation.

## Ethics Statement

The studies involving human participants were reviewed and approved by Loyola University Chicago Institutional Review Board. Written informed consent to participate in this study was provided by the participant or the participants’ legal guardian/next of kin.

## Author Contributions

DO and TK contributed to conception and design of the study and performed the statistical analysis. OC and KS performed integral literature reviews. DO, OC, TK, KS, and GH wrote sections of the manuscript. GH, OC, and KS edited the manuscript. MS organized the database. All authors contributed to manuscript revision, read, and approved the submitted version.

## Conflict of Interest

The authors declare that the research was conducted in the absence of any commercial or financial relationships that could be construed as a potential conflict of interest.

## Publisher’s Note

All claims expressed in this article are solely those of the authors and do not necessarily represent those of their affiliated organizations, or those of the publisher, the editors and the reviewers. Any product that may be evaluated in this article, or claim that may be made by its manufacturer, is not guaranteed or endorsed by the publisher.
